# Chest radiography patterns in 75 adolescents with vertically-acquired human immunodeficiency virus (HIV) infection

**DOI:** 10.1016/j.crad.2010.10.009

**Published:** 2011-03

**Authors:** S.R. Desai, S.J. Copley, R.D. Barker, C.M. Elston, R.F. Miller, A.U. Wells, S. Munyati, K. Nathoo, E.L. Corbett, R.A. Ferrand

**Affiliations:** a King’s College London, King’s Health Partners, Department of Radiology, King’s College Hospital NHS Foundation Trust, UK; b Department of Radiology, Hammersmith Hospital, UK; c King's College London, King’s Health Partners, Department of Respiratory Medicine, King’s College Hospital NHS Foundation Trust, UK; d Research Department of Infection and Public Health, Division of Population Health, University College London, UK; e Clinical Research Unit, London School of Hygiene and Tropical Medicine, London, UK; f The Interstitial Lung Disease Unit, Royal Brompton and Harefield NHS Trust, London, UK; g Biomedical Research and Training Institute, Samora Machel Avenue, Zimbabwe; h Department of Paediatrics, University of Zimbabwe, Zimbabwe; i Harare Central Hospital, Lobengula Road, Harare, Zimbabwe

## Abstract

**Aim:**

To evaluate lung disease on chest radiography (CR), the relative frequency of CR abnormalities, and their clinical correlates in adolescents with vertically-acquired human immunodeficiency virus (HIV) infection.

**Materials and methods:**

CRs of 75 patients [59 inpatients (33 males; mean age 13.7 ± 2.3 years) and 16 outpatients (eight males; mean age 14.1 ± 2.1 years)] were retrospectively reviewed by three independent observers. The overall extent of disease (to the nearest 5%), its distribution, and the proportional extents (totalling 100%) of different radiographic patterns (including ring/tramline opacities and consolidation) were quantified. CR features and clinical data were compared.

**Results:**

CRs were abnormal in 51/75 (68%) with “extensive” disease in 38/51 (74%). Ring/tramline opacities and consolidation predominated (i.e., proportional extent >50%) in 26 and 21 patients, respectively. Consolidation was significantly more common in patients hospitalized primarily for a respiratory illness than patients hospitalized for a non-respiratory illness or in outpatients (*p* < 0.005, χ^2^ for trend); by contrast, ring/tramline opacities did not differ in prevalence across the groups. On stepwise logistic regression, predominant consolidation was associated with progressive dyspnoea [odds ratio (OR) 5.60; 95% confidence intervals (CI): 1.60, 20.1; *p* < 0.01] and was associated with a primary respiratory cause for hospital admission (OR: 22.0; CI: 2.7, 181.1; *p* < 0.005). Ring/tramline opacities were equally prevalent in patients with and without chronic symptoms and in those admitted to hospital with respiratory and non-respiratory illness.

**Conclusion:**

In HIV-infected adolescents, evaluated in secondary practice, CR abnormalities are prevalent. The presence of ring/tramline opacities, believed to reflect chronic airway disease, is not linked chronic respiratory symptoms.

## Introduction

Southern Africa has borne the brunt of the human immunodeficiency virus (HIV) epidemic; the adult prevalence in most countries of this region exceeds 20%.^1^ In the absence of intervention, the rate of mother-to-child HIV transmission is around 30%.^2^ Thus, the adult epidemic has been followed by a regional epidemic of paediatric HIV/acquired immunodeficiency syndrome. Although untreated HIV infection in infants is associated with a high risk of early disease progression and death,^3^ up to one-third of infants are believed to progress slowly and at least 10% may survive into adolescence.^4–6^ In children with HIV infection, lung disease appears to be common. An entity of “chronic lung disease” is recognized,^7–11^ but its causes and characteristics have yet to be fully defined. A high prevalence of chronic lung disease on chest radiography has been identified in young children,^10^ but this cannot be extrapolated to older children who, conceivably, might survive to adolescence by virtue of a milder disease phenotype.

The aim of the present study was to determine whether chest radiography provides potentially important information in the evaluation of HIV adolescents, both in routine practice, and in future population studies of chronic lung disease. Specifically, we have evaluated the prevalence, nature, and clinical correlates of chest radiographic abnormalities in adolescents with HIV infection in Zimbabwe, managed in a hospital practice.

## Materials and methods

### Patient population

Eighty HIV-infected adolescents (aged 10 to 18 years) infected through mother-to-child transmission were recruited; a subset (*n* = 59) of patients has been reported in two separate studies of skin disease^12^ and causes of acute hospitalization^13^ in HIV, neither of which dealt with chest radiographic features. Patients were either attending an outpatient HIV care clinic or had been admitted to one of two public-sector hospitals in Harare, Zimbabwe, because of fever and/or respiratory symptoms; hospitalized patients were subcategorized as having (1) a primary respiratory cause or (2) primary non-respiratory cause for admission. The first chest radiographs of patients (CR; obtained in the previous 6 months as part of routine HIV outpatient care or the first radiograph at or immediately following hospital admission) were reviewed; the CRs of five patients were of suboptimal quality and excluded.

Vertical HIV acquisition was considered the most likely mode by the study physician, based on clinical evaluation, history (growth failure, longstanding ill-health, maternal and/or sibling HIV infection) and no reported risk of sexual or parenteral exposure to HIV infection. Accurate demographic and clinical data were available in 59 hospital inpatients: clinical records were reviewed with specific reference to the presence of the following chronic respiratory symptoms (present for 3 months of the year in the last two years): (1) a non-productive cough, (2) a cough productive of sputum, and (3) a history of progressive breathlessness over the previous 3 months. The primary cause (respiratory versus non-respiratory) for admission and the numbers receiving highly-active antiretroviral therapy (HAART) were recorded. The study was approved by the Medical Research Council of Zimbabwe and the Institutional Review Boards of the Biomedical Research and Training Institute (Harare), The London School of Hygiene and Tropical Medicine (London), and the Institutional Review Boards at both participating public-sector hospitals (Harare). Informed consent was obtained from all inpatients but the need for consent from outpatients was waived.

### Chest radiographic scoring

CRs were reviewed retrospectively by three independent observers blinded to clinical data. The observers were two thoracic radiologists (S.R.D., S.J.C., both subspecialty-trained, in clinical practice at teaching institutions in the UK and with >10 years experience in thoracic imaging) and a consultant chest physician (R.D.B. with 15 years clinical experience in pulmonary medicine but no prior experience of quantitative radiographic evaluation). Chest radiographic scoring was adapted from methods previously reported^4,16^ but observations were confined to standardized radiographic terminology.^14^ The scoring system included an evaluation not only of the presence/absence of radiographic signs, but also the overall and proportional extents of chest radiographic patterns and the cranio-caudal distribution of disease. The following radiographic features were quantified and/or recorded: (1) the overall extent of abnormal lung was graded semi-quantitatively on a five-point scale (0 = normal; 1 = 1–25% abnormal lung; 2 = 26–50%; 3 = 51–75% and 4 = 76–100%). For quantification purposes, each lung projected on the CR was considered to represent 50% of the total. (2) The presence or absence of the following radiographic patterns (hereafter termed the “principal” patterns) was recorded: (a) consolidation (defined as an homogeneous increase in pulmonary parenchymal attenuation that obscures the margins of vessels and airway walls ^14^); (b) reticular pattern (defined as a collection of innumerable small linear opacities that, by summation, produce an appearance resembling a net); (c) non-cavitating nodules (defined as a rounded opacity or opacities, well or poorly defined, measuring up to 3 cm in diameter; (d) cavitating nodules (a cavity was defined as a gas-filled space, seen as a lucency or low-attenuation area within a nodule; (e) ring/tramline opacities: believed to reflect airway abnormalities (i.e., bronchial wall thickening and/or bronchiectasis); (f) ground-glass opacification (defined as an area of hazy increased lung opacity within which margins of pulmonary vessels may be indistinct); and (g) masses (defined as any pulmonary lesion seen on CRs as an opacity greater than 3 cm in diameter). (3) The proportional extents of the principal radiographic patterns were quantified to the nearest 5% with the total extent (where more than one pattern was present) not exceeding 100%. (4) The cranio-caudal (upper, mid and/or lower) distribution of abnormalities was recorded: the upper-zone was defined as that region of the projected lung on a CR above the upper margin of the anterior second rib. The mid-zone was that region between the lower margin of the anterior second rib and the upper margin of the anterior fourth rib and the lower zone was defined as that region below the lower margin of the fourth rib.

The presence/absence of the following radiographic abnormalities (termed the “ancillary” patterns) was also recorded: (1) bullae; (2) paucity of vessels; (3) pleural effusion; (4) hilar and/or mediastinal lymph node enlargement; and (v) volume loss.

Discrepant observations were resolved by consensus review between observers: a difference in the overall extent of abnormal lung of greater than one grade, between any two observers, was resolved by consensus. Discrepant observations for the presence or absence of the principal radiographic patterns and their distribution were also resolved; a difference in the proportional extents of the principal patterns exceeding 20% was the threshold for consensus review. Similarly, discrepant observations for the presence or absence of ancillary radiographic findings were resolved by consensus.

### Statistical analysis

Data were analysed using STATA (version 9.0; StataCorp, College Station, TX, USA), and are expressed as mean ± standard deviation for continuous variables and proportions for categorical variables; *p* < 0.05 was taken as the threshold for statistical significance. Observer variation for quantifying the overall extent of abnormal lung, determining the zonal distribution of disease and the proportional extent of individual radiographic patterns was expressed as the kappa coefficient [weighted or unweighted (as appropriate)]. Univariable correlations were examined using the Spearman’s correlation coefficient or Pearson’s product-moment correlation coefficient (as appropriate). Differences between proportions were tested using the χ^2^ test or Fisher’s exact test (as appropriate). Comparisons between groups were tested using χ^2^ for trend. Stepwise logistic regression models were constructed to determine the independent relationships between radiographic patterns and clinical parameters.

## Results

### Baseline demographic and clinical data

CRs of 75 adolescents [59 hospital inpatients (M:F 33:26; mean age 13.7 ± 2.3 years) and 16 outpatients (M:F 8:8; mean age 14.1 ± 2.1 years)] were reviewed. In patients admitted to hospital, the principal cause for admission was a respiratory illness in 36/59 (61%). Hospitalized patients reported chronic symptoms as follows: (a) a cough for 3 months of the year in the last 2 years in 16/59 (27%) patients, (b) a cough productive of sputum for 3 months of the year in the last 2 years in 8/59 (14%) patients; and (c) a history of progressive breathlessness over the past 3 months in 31/59 (52%) patients. Twenty-one of 59 (36%) patients were taking HAART.

### Baseline chest radiographic observations

CRs were abnormal in 51/75 (68%) patients. Disease was of limited extent (summed extent grade for three observers ≤3) in 13/51 (26%) and extensive (summed grades 4–12) in 38/51 (74%) of patients. The distribution of disease was as follows: right upper, mid and lower zones in 12/51, 26/51, and 37/51 patients, respectively, and left upper mid and lower zones in 11/51, 31/51, and 39/51 patients, respectively. Thus, lung disease was most common in the mid and lower zones.

### Prevalence of principal and ancillary chest radiographic patterns

**Figure 1:**
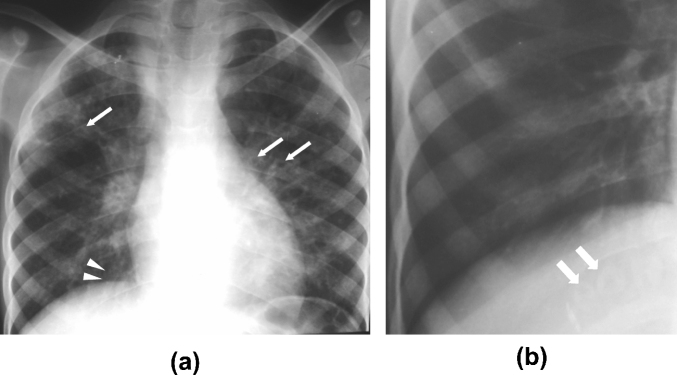
CRs in two patients with vertically-acquired HIV infection. (a) 16-year-old male patient with rings (arrows) and tramline opacities (arrowheads) in both lungs, principally in the mid- and upper zones, and (b) targeted image of the right lower zone showing ring opacities (taken to indicate thick-walled, bronchiectatic airways).

**Figure 2:**
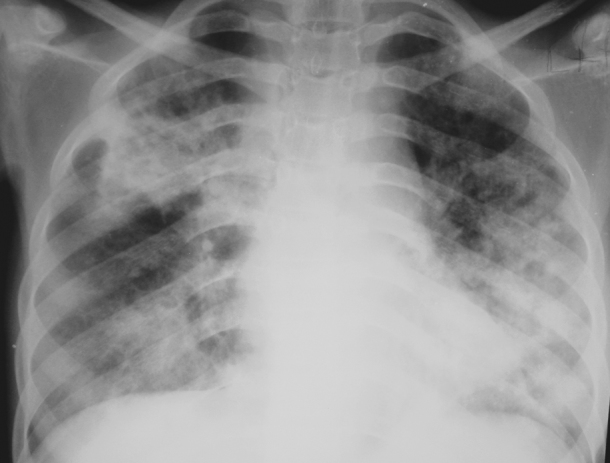
CR in a 14-year-old HIV-positive adolescent with predominant and patchy bilateral consolidation.

Ring/tramline opacities [seen in 42/75 (56%) patients] and consolidation [present in 29/75 (39%)] were the most prevalent CR patterns (Figs 1 and 2). Ring/tramline opacities co-existed with consolidation in 21/75 (28%) patients. A reticular pattern, nodules (cavitating and non-cavitating), ground-glass opacification, and masses were present in less than 15% of patients. Ancillary CR features included a paucity of vessels [15/75 (20%)], pleural effusion [8/75 (11%)], hilar and/or mediastinal lymph node enlargement [9/75 (12%)] and volume loss [6/75 (8%)].

There were predominant (i.e., proportional extent ≥50%) ring/tramline opacities in 26 patients either as the most extensive pattern (*n* = 20) or equally admixed with non-cavitating nodules (*n* = 3), consolidation (*n* = 2), or ground-glass opacification (*n* = 1). Consolidation predominated in 21 patients (in isolation, *n* = 19) or was equally admixed with ring/tramline opacities (*n* = 2). Non-cavitating nodules predominated in six patients (in isolation, *n* = 3 and equally predominant with ring/tramline opacities, *n* = 3) and ground glass opacification in four patients (in isolation, *n* = 3 and equally predominant with ring/tramline opacities, *n* = 1). There was no patients with an intrapulmonary mass.

### Observer variation

Observer agreement for the evaluation of CR features was expressed as the kappa coefficient (weighted or unweighted, as appropriate; Table 1).^15^ There was good agreement between the three observers for quantification of the overall extent of lung disease (weighted kappa coefficient, Kω = 0.87–0.91).^16^ Agreement for evaluating the zonal presence of disease was also above the clinically acceptable range (unweighted K = 0.57–0.80). Observer variation for the quantification of proportional extents of the principal CR patterns was also above clinically acceptable limits (Table 1) except the observer agreement for non-cavitating nodules (K_w_ = 0.26).^16^ A reticular pattern (*n* = 2), cavitating nodules (*n* = 1), ground-glass opacities (*n* = 5), and masses (*n* = 0) were present in too few patients and observer variation was not quantified.

### Relationships between principal and ancillary radiographic patterns

Consolidation was present in 21/42 patients with ring/tramline opacities, but in only 8/33 patients without ring/tramline opacities (*p* = 0.02). Non-cavitating nodules were present in 18/42 patients with ring/tramline opacities, but in only 2/33 without ring/tramline opacities (*p* < 0.0005). There were no other significant associations between the principal CR patterns.

Hilar and/or mediastinal lymph node enlargement was seen in 7/29 patients with consolidation but in only 2/46 without consolidation (*p* = 0.02). Similarly, hilar and/or mediastinal lymph node enlargement was identified in 9/42 patients with ring/tramline opacities and in none of the patients without ring/tramline opacities (*p* < 0.005). There was hilar and/or mediastinal lymph node enlargement in 7/21 patients with co-existent ring/tramline opacities and consolidation but in only 2/54 without combined ring/tramline opacities and consolidation (*p* < 0.01). There were no other significant relationships between the principal and ancillary radiographic features.

### Comparison between inpatient subgroups and outpatients

The prevalence of consolidation and ring/tramline opacities was examined in the three subgroups: (a) patients hospitalized primarily for respiratory cause (*n* = 36); (b) patients hospitalized primarily for a non-respiratory cause (*n* = 23); and (c) patients attending the outpatient HIV care clinic (*n* = 16). As shown in Table 2, consolidation was strikingly more prevalent in patients hospitalized with a respiratory illness than in the other two groups (*p* < 0.005, χ^2^ for trend). In contrast, ring/tramline opacities did not differ significantly in prevalence across the three groups. However, when the two inpatient groups were combined ring/tramline opacities were more prevalent in hospitalized patients [36/59 (61%)] than in the outpatient subgroup [6/16 (38%); *p*<0.005].

### Relationships between radiographic patterns and clinical data in 59 inpatients

A pattern of predominant consolidation on CR was linked with a history of progressive breathlessness over the past 3 months (odds ratio, OR = 5.60; confidence intervals, CI = 1.60, 20.1; *p* < 0.01). A pattern of predominant consolidation was also strongly associated with a primary respiratory cause for admission to hospital as compared to a non-respiratory cause for admission (OR = 22; CI = 2.7, 181.1; *p* < 0.005). There was no independent association between predominant consolidation on CR and treatment with HAART.

The presence of ring/tramline opacities was not linked to the presence of chronic symptoms, a respiratory indication for hospital admission or treatment with HAART (see Table 3). Similarly, there were no independent associations between other chest radiographic abnormalities and chronic pulmonary symptoms, a primary respiratory cause for hospital admission or treatment with HAART.

## Discussion

Lung disease is believed to be common among HIV-infected children^7,8,10,11,17^ but, to the authors’ knowledge, this is the first study to evaluate CR patterns in adolescents with vertically-acquired HIV infection. The findings of the present study indicate that abnormalities are prevalent on CR; ring/tramline opacities and consolidation were the most common patterns. Consolidation was strongly associated with a respiratory admission to hospital. However, rings/tramline opacities (the predominant radiographic pattern) did not differ significantly in prevalence between patient subgroups, suggesting that this radiographic pattern might represent “chronic” lung disease. Moreover, the prevalence of ring/tramline opacities did not differ between patients with and without chronic symptoms. The present findings have a potential bearing on clinical management and future studies in this area: it seems likely that the presence or absence or chronic respiratory symptoms will not differentiate between patients with and without radiographic evidence of possible chronic lung disease. Our results also suggest that chest radiography should be integrated with routine clinical evaluation in HIV-infected adolescents with suspected lung disease, chronic or otherwise.

Only a few studies have formally evaluated CR abnormalities in HIV-positive children^8,10^ and then, only in younger cohorts. In one study, of 28 HIV-infected children with a mean age of 3 years, persistent reticulonodular opacities were present in just over 50%.^8^ In another study, of children with a median age of just under 2 years, consolidation, increased bronchovascular markings and reticulation and/or nodules were present in roughly one-third of a subgroup of 201 patients.^10^ The most common chronic patterns, seen in 20% in this study, were an increase in bronchovascular markings or reticular densities. In contrast, the most prevalent “transient” or acute radiographic patterns were consolidation and increased interstitial markings.^10^

When considering earlier chest radiographic series, issues related to the grouping of signs at analysis, the use of non-standard radiological terminology, and observer disagreement should not be overlooked. In the study by Norton and colleagues, bronchovascular markings and reticular densities were combined because of observer-related “noise”.^10^ This approach is problematic because different radiographic patterns are likely to reflect variable pathological processes. The use of non-conventional radiographic terms is also an issue because terms, such as “dense patchy opacities”, “interstitial markings”, and “patchy opacification”, lack precision and are open to broad interpretation. There are two other concerns that apply to chest radiographic observations in general: first, differences in perception, and second, the level of observer experience.^18–20^ These factors can contribute to inter- and intra-observer variability.

In the present study, observations were confined to the accepted definitions of radiographic patterns.^14^ Furthermore, the radiographic patterns were evaluated and analysed separately. There are two further points in support of the radiological methods in the present study: first, observer disagreement for the key radiological signs was in the clinically-acceptable range. Second, the readings of the non-radiologist, with no previous experience of radiological scoring, accorded closely with those of two thoracic radiologists, indicating that the system is likely to be both applicable and robust for physicians involved in the management of adolescents with HIV-related lung disease.

Without serial chest radiography or high-resolution computed tomography (HRCT), the extrapolation of plain radiographic patterns to the distinction between acute and chronic disease must be made with caution. However, the previously reported linkages between consolidation and acute disease,^10^ and between ring shadows or increased “bronchovascular markings” (broadly comparable to the ring/tramline opacities in the present study) and chronic disease^8,10^ are supported by the subgroup findings in the present study: consolidation was seldom present except in patients hospitalized with respiratory disease. In contrast, the similar prevalence of ring/tramline opacities in all patient subgroups and the lack of a linkage with acute respiratory illness suggest that this radiographic pattern is more likely to represent chronic disease. However, the important limitations of the present study must be mentioned. First, there is the potential for bias due to the retrospective design. Second, the relatively small numbers in the patient subgroups (particularly outpatients) is problematic as it could reasonably be argued that CRs in outpatients might have inadvertently “selected” patients with more acute (but not hospitalized) respiratory illness. These issues highlight the need for prospective studies of chronic lung disease in HIV-infected adolescents with serial chest radiography or, indeed, HRCT at their core.

The morphological basis of chronic lung disease in paediatric HIV infection is still debated. However, lymphocytic pulmonary infiltration, infection, and bronchiectasis are believed to be contributory.^7–11^ The ring/tramline opacities may be a manifestation of airway inflammation reflecting bronchial wall thickening and/or bronchiectasis. As regards the latter, there is an acknowledged link between bronchiectasis and HIV-infection^7,21–23^ with recurrent bacterial infections^7,23^ or HIV-infection *per se*, believed to play an aetiological role.^22^ However, with respect to bronchiectasis specifically, it should be noted that chest radiographic diagnosis is not straightforward, even for experienced observers: the lower sensitivity of chest radiography (particularly for mild disease) and the notoriously non-specific radiographic appearances are important limitations.^24–26^ Again, in this regard, HRCT is likely to play a key role in future studies to define the morphological basis of chronic lung disease in this group of patients.

Given that the majority of patients in this study were inpatients, acute changes (characterized by consolidation) might have been expected to have been the commonest pattern; yet consolidation was only present in a minority of cases with ring/tramline opacities predominating. From the above, it is reasonable to conclude that larger population-based studies to identify chronic disease will be informative. However, here again, the current study provides an insight: the frequency of ring/tramline opacities did not differ between patients with and without chronic symptoms. It follows that in future population-based studies, the presence of chronic symptoms cannot be considered an accurate surrogate for HIV-related lung disease.

In summary, CR abnormalities are highly prevalent in adolescents with vertically-acquired HIV infection evaluated in secondary practice. The presence of ring/tramline opacities (the most prevalent radiographic pattern, which may represent chronic lung disease) is not related to respiratory symptoms. These findings highlight the potential importance of chest radiography and the limitations of clinical evaluation alone, both in routine management and in future studies of HIV-related lung disease.

## Figures and Tables

**Table 1:** Observer variation between three readers for quantification of disease extent, determination of zonal distribution, and scoring of individual radiographic patterns

Chest radiographic parameter	Kappa coefficient (weighted, Kω / unweighted, K)
Overall extent of disease	
Observer 1 versus 2	Kω = 0.88
Observer 1 versus 3	Kω = 0.87
Observer 2 versus 3	Kω = 0.91
Zonal disease distribution	
Upper zone	K = 0.80
Mid zone	K = 0.57
Lower zone	K = 0.69
Extent of radiographic pattern	
Consolidation	K = 0.70
Reticular pattern	N/A
Non-cavitating nodules	Kω = 0.26
Cavitating nodules	N/A
Ring/tramline opacities	K = 0.51
Ground-glass opacities	N/A
Masses	N/A

**Table 2:** Comparison of the prevalence of consolidation and ring/tramline opacities between patient subgroups

	In patients		Outpatients (*n* = 16)	*p*-value
	Respiratory (*n* = 36)	Non-respiratory (*n* = 23)		
Consolidation	21/36 (58%)	4/23 (17%)	4/16 (25%)	<0.005
Ring/tramlines	21/36 (58%)	15/23 (65%)	6/16 (38%)	0.21^a^

a Ring/tramline opacities were significantly more prevalent in the combined group of hospitalized patients [36/59 (61%)] than in outpatients [6/16 (38%); *p*<0.005].

**Table 3:** Comparison of the prevalences of ring/tramline opacities in 59 inpatients with and without (1) a chronic cough, (2) a cough productive of sputum, (3) progressive dyspnoea, (4) a primary respiratory cause for admission and (5) a history of treatment with HAART

	Clinical feature present	Clinical feature absent	*p*-value
Cough (*n* = 16)	11/16 (69%)	25/43 (58%)	0.46
Productive cough (*n* = 8)	5/8 (62%)	31/51 (61%)	0.93
Progressive dyspnoea (*n* = 31)	21/31 (68%)	15/28 (54%)	0.27
Respiratory admission (*n* = 36)	21/36 (58%)	15/23 (65%)	0.60
HAART therapy (n = 21)	12/21 (57%)	24/38 (63%)	0.65

The table describes the prevalence of ring/tramline opacities in patients with and without specific clinical features; for example, ring/tramline opacities were present in 11/16 (69%) patients with a chronic cough but also in 25/43 (58%) patients without a chronic cough. HAART, highly-active antiretroviral therapy.
